# Author Correction: Cancer genomes tolerate deleterious coding mutations through somatic copy number amplifications of wild-type regions

**DOI:** 10.1038/s41467-023-40060-z

**Published:** 2023-07-21

**Authors:** Fabio Alfieri, Giulio Caravagna, Martin H. Schaefer

**Affiliations:** 1 Department of Experimental Oncology, IEO European Institute of Oncology IRCCS, 20139 Milan, Italy; 2 Department of Mathematics and Geosciences, University of Trieste, 34127 Trieste, Italy

**Keywords:** Computational biology and bioinformatics, Tumour heterogeneity, Cancer genomics, Gene amplification, Evolutionary theory

Correction to: *Nature Communications* 10.1038/s41467-023-39313-8, published online 16 June 2023

The original version of this Article contained errors in Figs. 1b, 4a, and 5c. In Fig. 1b, the *y*-axis had wrong interval distances between the ticks, specifically between 0.1 and 0.2. In Fig. 4a, in the “non-coding” (blue) 2:1 segment specific copy number bar, the numbers indicating the numerosity were swapped between “late” (dark blue) and “early” (light blue). In Fig. 5c, the text of the legend was inadvertently omitted. These errors have been corrected in both the PDF and HTML versions of the Article.

