# Supplementary material for: Cancer genomes tolerate deleterious coding mutations through somatic copy number amplifications of wild-type regions
Source: Nat Commun. 2023 Jun 16;14:3594. doi: 10.1038/s41467-023-39313-8 (PMC10276008; doi:10.1038/s41467-023-39313-8)
Supplement: Supplementary file 1 — Supplementary Information [file 41467_2023_39313_MOESM1_ESM.pdf]

Supplementary Information for

Cancer genomes tolerate deleterious coding mutations through somatic copy number amplifications of wild-type regions

Fabio Alfieri[1], Giulio Caravagna[2], Martin H. Schaefer[1,*]

*** Corresponding author**

martin.schaefer@ieo.it (M.S.)

The PDF file includes:

- Supplementary Dataset legends (1 to 4)
- Supplementary Figures (1 to 9)

Supplementary Dataset legends

Supplementary Dataset 1

μ score and amplification frequency for each tumor type at 1Mbp, 36Mbp and arm-level.

Supplementary Dataset 2

Aggregation estimates computed with TANGO. The "Aggregation" column represents the raw scores computed by TANGO; the FoldChange column is computed using mutant aggregation score over the wild-type aggregation score.

Supplementary Dataset 3

Pancancer protected and unprotected genes. Gene Ontology results of pancancer protected and unprotected gene sets, multiple testing correction performed. P_i stands for protection index. CRISPR common essential and non-essential genes.

Supplementary Dataset 4

Number of genes and mutations in each subset of Fig. 2.

Supplementary Figures

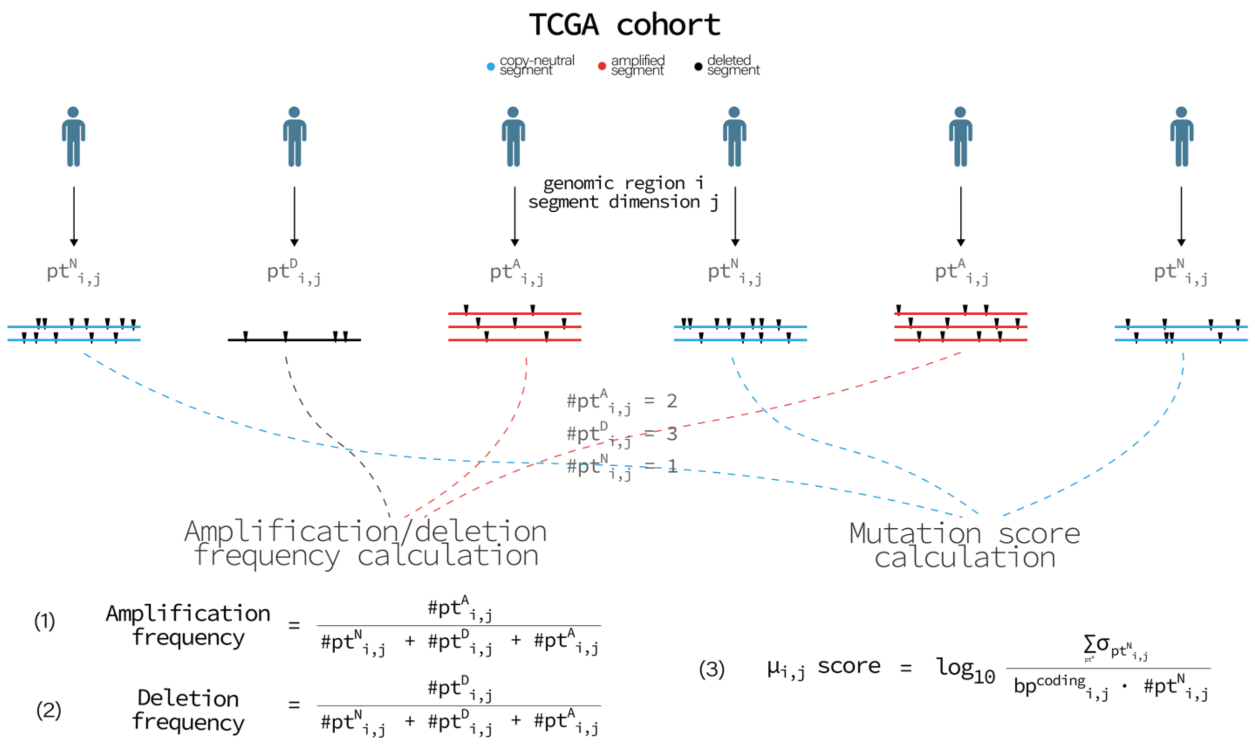

Figure S1

A cartoon illustration of the method used for calculating the mutation score (the $\mu_{i,j}$ score) and the amplification/deletion frequencies. After the classification of segments (i) with a specific length (j) for each patient ($pt_{i,j}$) in copy-neutral ($pt_{i,j}^{N}$), amplified ($pt_{i,j}^{A}$) or deleted ($pt_{i,j}^{D}$) based on their copy number, the amplification frequency is calculated as the number of patients with an amplified segment ($\#pt_{i,j}^{A}$) over the total number of patients ($\#pt_{i,j}^{N} + \#pt_{i,j}^{A} + \#pt_{i,j}^{D}$) within the analyzed TCGA cohort (1) and (2). The μ score (3) is calculated by summing mutations only within the copy-neutral segments ($\sigma_{pt_{i,j}^{N}}$) and thus normalizing for the number of copy-neutral patients ($pt_{i,j}^{N}$) and the number of protein-coding nucleotides within the segment i ($bp_{i,j}^{coding}$). Human being representations were adapted from “Icon Pack - Human Icons” by BioRender.com, retrieved from https://app.biorender.com/biorender-templates/.

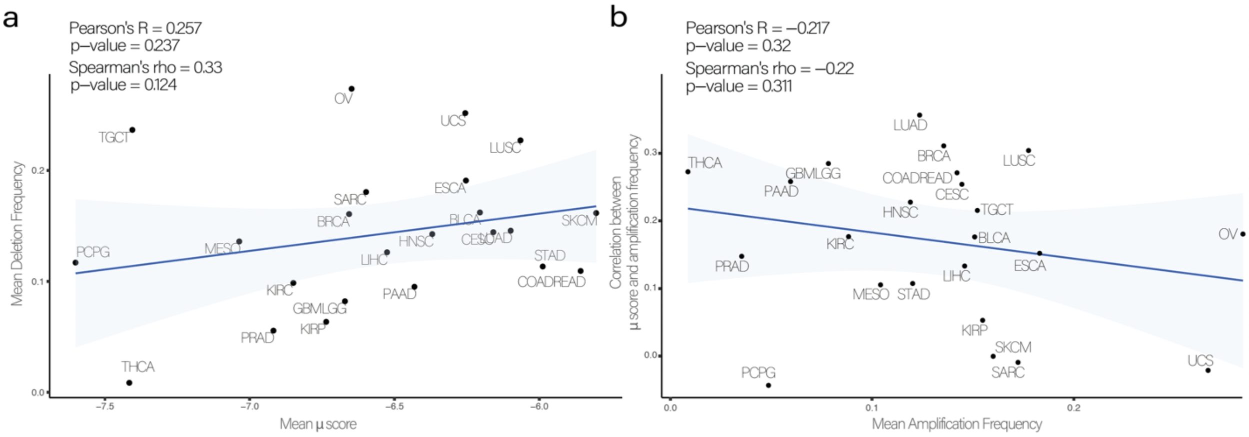

Figure S2

The error bands reflect the 95% confidence intervals of these estimates.

a: Correlation between mean mutation (μ) score and mean deletion frequency calculated using Spearman's test.

b: Correlation between mean amplification frequency and Spearman's correlations (between μ score and amplification frequencies) calculated using Spearman's test.

Lung Squamous cell Carcinoma (LUSC); Lung Adenocarcinoma (LUAD); Colon Adenocarcinoma and Rectum Adenocarcinoma (COADREAD); Cervical Squamous cell Carcinoma and Endocervical Carcinoma (CESC); Breast invasive Carcinoma (BRCA); Skin Cutaneous Melanoma (SKCM); Ovarian Serous Cystadenocarcinoma (OV); Uterine Carcinosarcoma (UCS); Liver Hepatocellular Carcinoma (LIHC); Head and Neck Squamous Carcinoma (HNSC); Prostate Adenocarcinoma (PRAD); Thyroid carcinoma (THCA); Pheochromocytoma and Paraganglioma (PCPG); Esophageal carcinoma (ESCA); Stomach adenocarcinoma (STAD); Glioblastoma multiforme, Brain Lower Grade Glioma (GBMLGG); Kidney renal clear cell carcinoma (KIRC); Kidney renal papillary cell carcinoma (KIRC); Pancreatic adenocarcinoma (PAAD); Testicular Germ Cell Tumors (TGCT); Mesothelioma, (MESO); Sarcoma (SARC); Bladder Urothelial Carcinoma (BLCA). Source data are provided as a Source Data file.

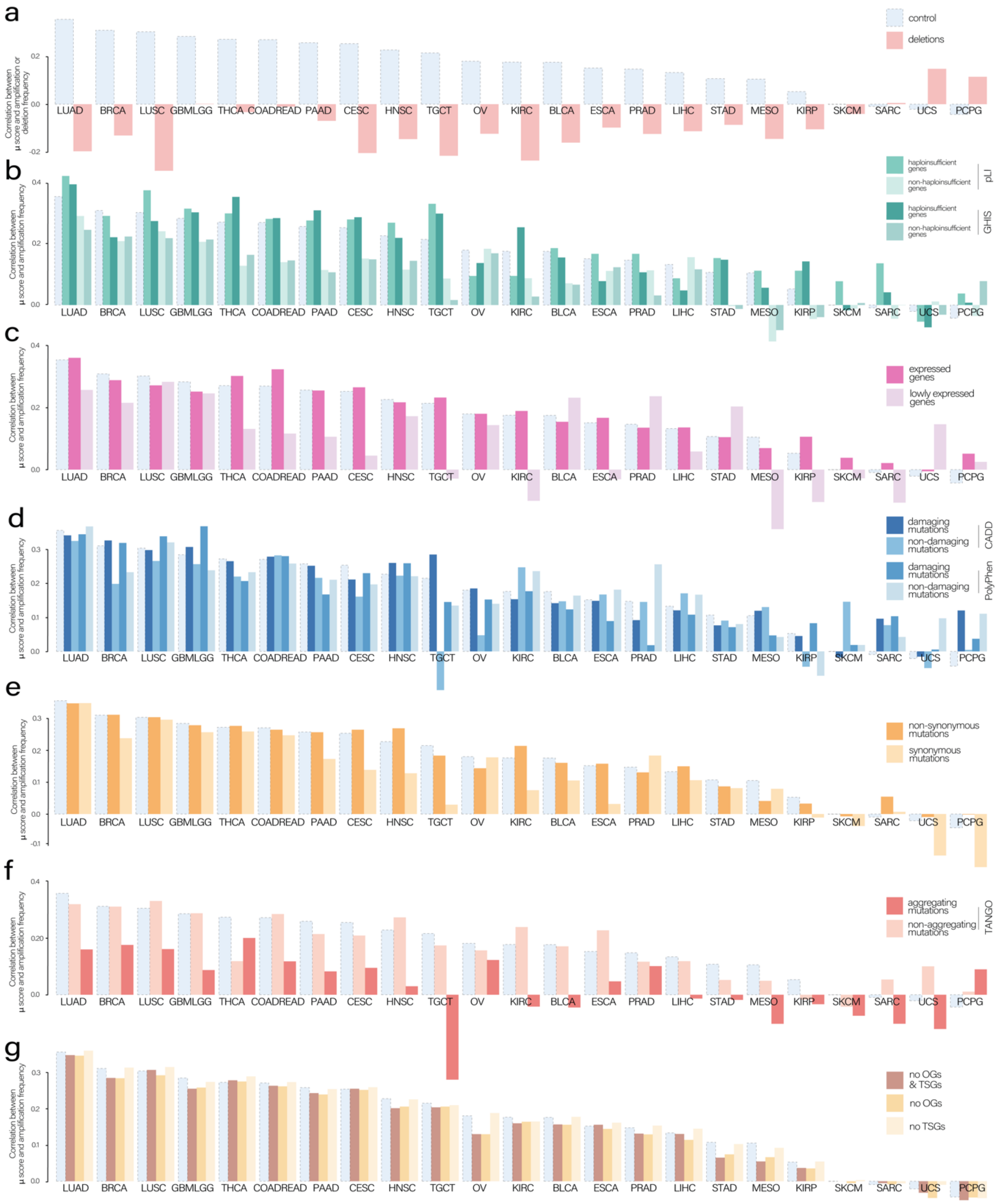

Figure S3

Extension of **Fig. 2** across all TCGA tumor types.

a: Correlations between the mutation (μ) score and amplification or deletion frequency across all TCGA tumor types.

b: Correlations between amplification frequency and μ score calculated using mutations within haploinsufficient and non-haploinsufficient genes.

c: Correlations between amplification frequency and μ score calculated using synonymous and non-synonymous mutations.

d: Correlations between amplification frequency and μ score calculated using aggregation-causing and non-aggregation-causing mutations.

e: Correlations between amplification frequency and μ score calculated using mutations within expressed and non-expressed genes.

f: Correlations between amplification frequency and μ score calculated using damaging and non-damaging mutations (predicted by CADD or Polyphen) mutations.

g: Correlations between amplification frequency and μ score calculated using mutations without OGs and TSGs, only without OGs or only without TSGs.

Lung Squamous cell Carcinoma (LUSC); Lung Adenocarcinoma (LUAD); Colon Adenocarcinoma and Rectum Adenocarcinoma (COADREAD); Cervical Squamous cell Carcinoma and Endocervical Carcinoma (CESC); Breast invasive Carcinoma (BRCA); Skin Cutaneous Melanoma (SKCM); Ovarian Serous Cystadenocarcinoma (OV); Uterine Carcinosarcoma (UCS); Liver Hepatocellular Carcinoma (LIHC); Head and Neck Squamous Carcinoma (HNSC); Prostate Adenocarcinoma (PRAD); Thyroid carcinoma (THCA); Pheochromocytoma and Paraganglioma (PCPG); Esophageal carcinoma (ESCA); Stomach adenocarcinoma (STAD); Glioblastoma multiforme, Brain Lower Grade Glioma (GBMLGG); Kidney renal clear cell carcinoma (KIRC); Kidney renal papillary cell carcinoma (KIRC); Pancreatic adenocarcinoma (PAAD); Testicular Germ Cell Tumors (TGCT); Mesothelioma, (MESO); Sarcoma (SARC); Bladder Urothelial Carcinoma (BLCA). Source data are provided as a Source Data file.

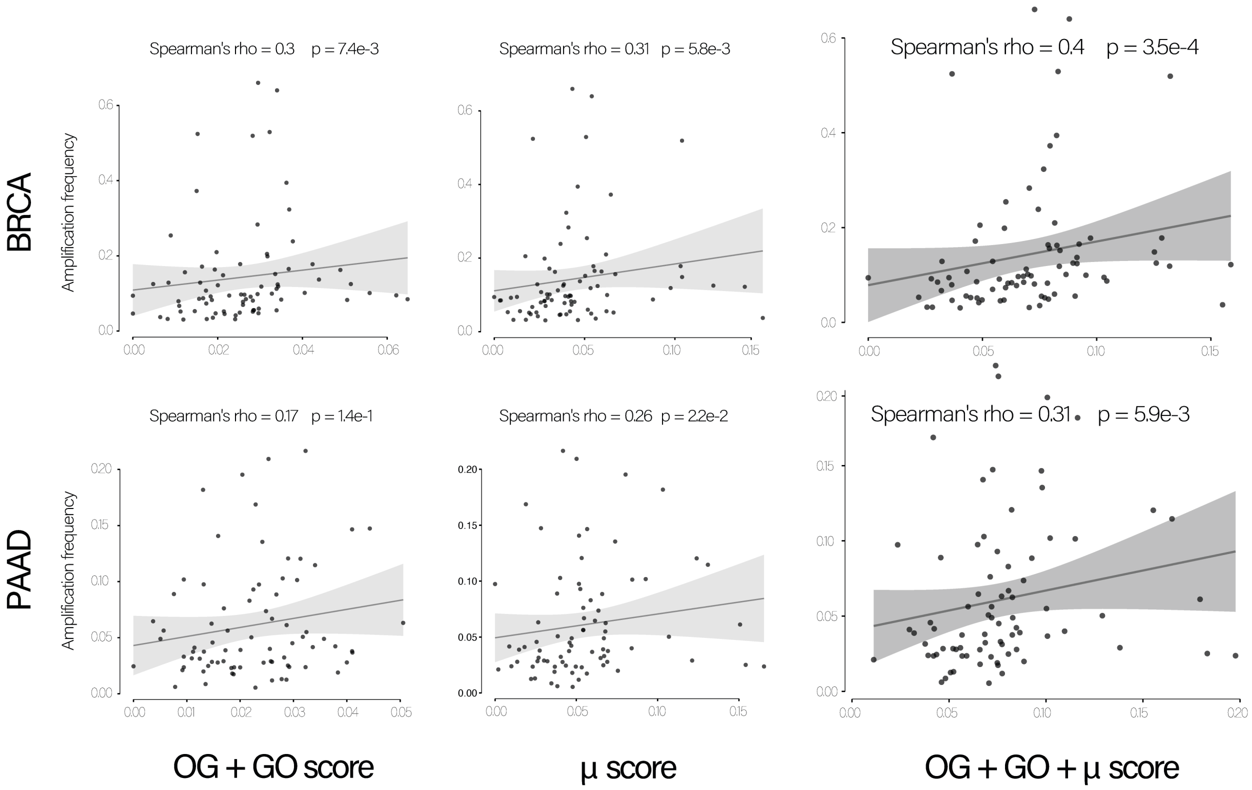

Figure S4

Correlations in Breast invasive Carcinoma (BRCA) and Pancreatic adenocarcinoma (PAAD) between amplification frequency and different predictors (figure refers to **Fig. 3**, controls are excluded) calculated using Spearman's test. The error bands reflect the 95% confidence intervals of these correlation estimates. Source data are provided as a Source Data file.

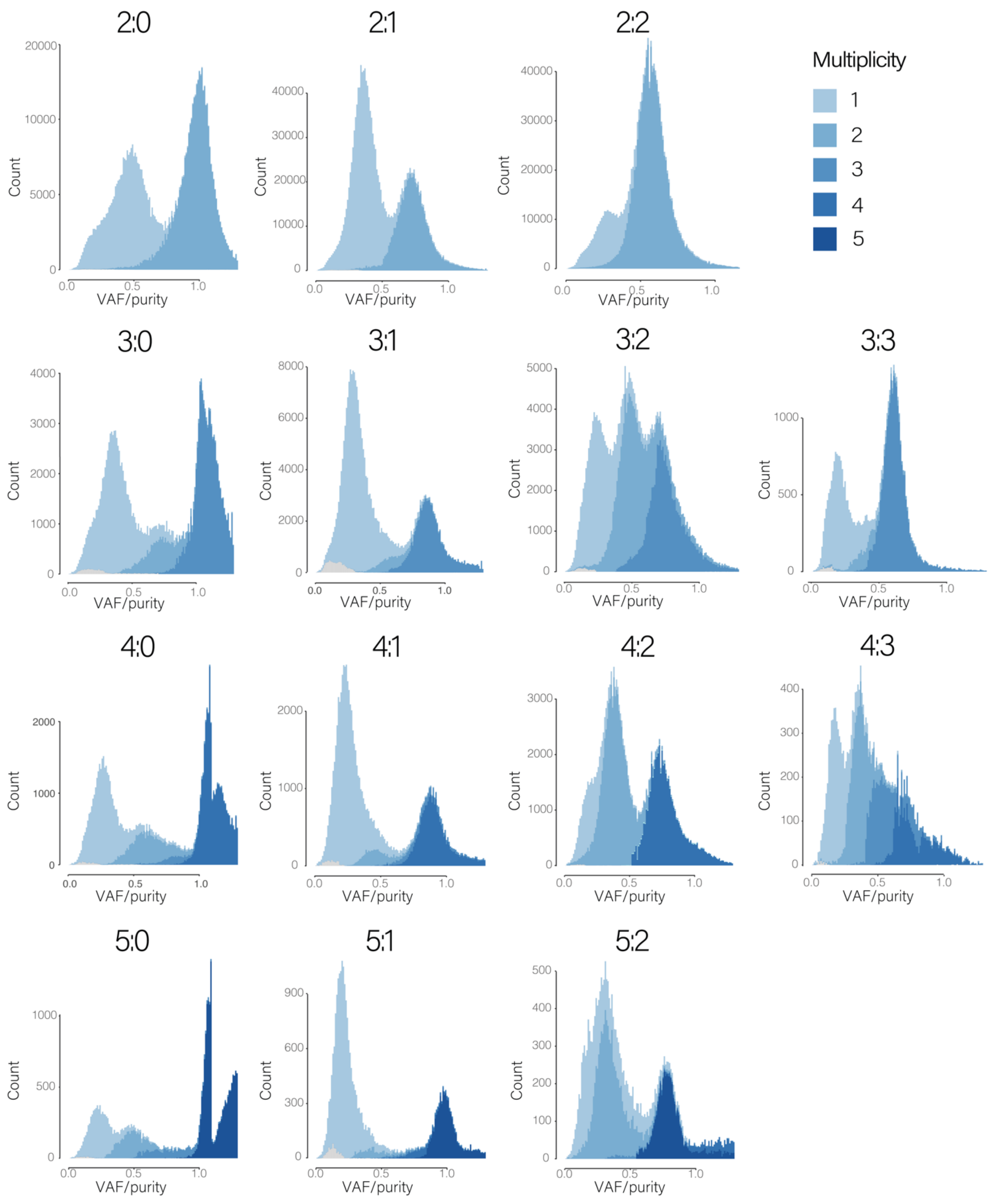

Figure S5

Histogram curves represent the different distribution of mutations according to VAF/purity, they are colored according to the predicted multiplicity. Above each histogram, X:Y represent the segment copy-number where X represents the major allele and Y represents the minor allele.

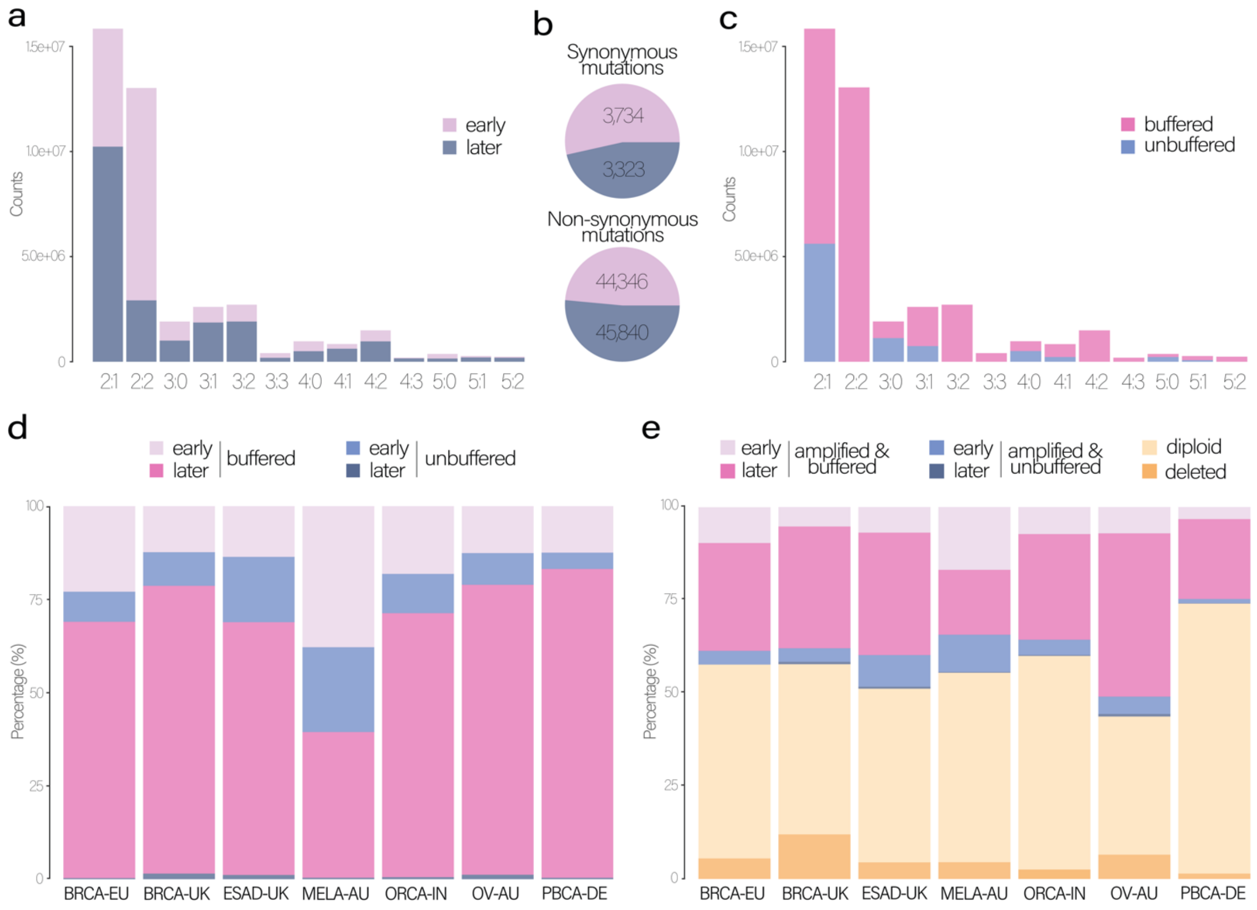

Figure S6

a: Mutations within amplifications are classified as early or late and shown across segment's allele-specific copy number.

b: Synonymous and non-synonymous coding mutations in the early or late classification.

c: Mutations within amplifications are classified as buffered or unbuffered and shown across segment's allele-specific copy-number.

d: Mutations within amplifications are classified as early or late and as buffered or unbuffered, simultaneously.

e: All mutations (deleted, diploid and amplified) are classified as early or late and buffered or unbuffered.

Breast cancer European Union (BRCA-EU), Breast cancer - United Kingdom (BRCA-UK), Esophageal Adenocarcinoma - United Kingdom (ESAD-UK), Skin cancer - Australia (MELA-AU), Oral cancer - India (ORCA-IN), Ovarian cancer – Australia (OV-AU), Pediatric Brain cancer – Germany (PBCA-DE). Source data are provided as a Source Data file.

a

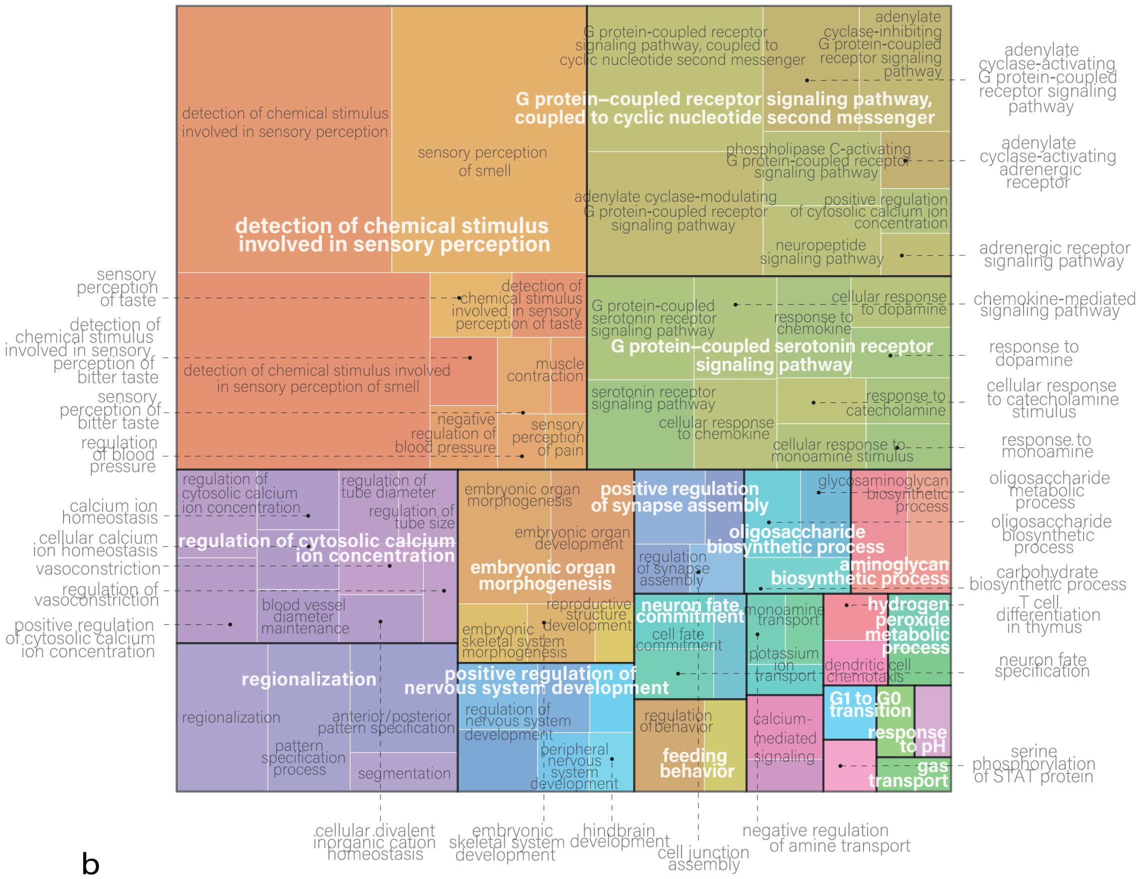

b

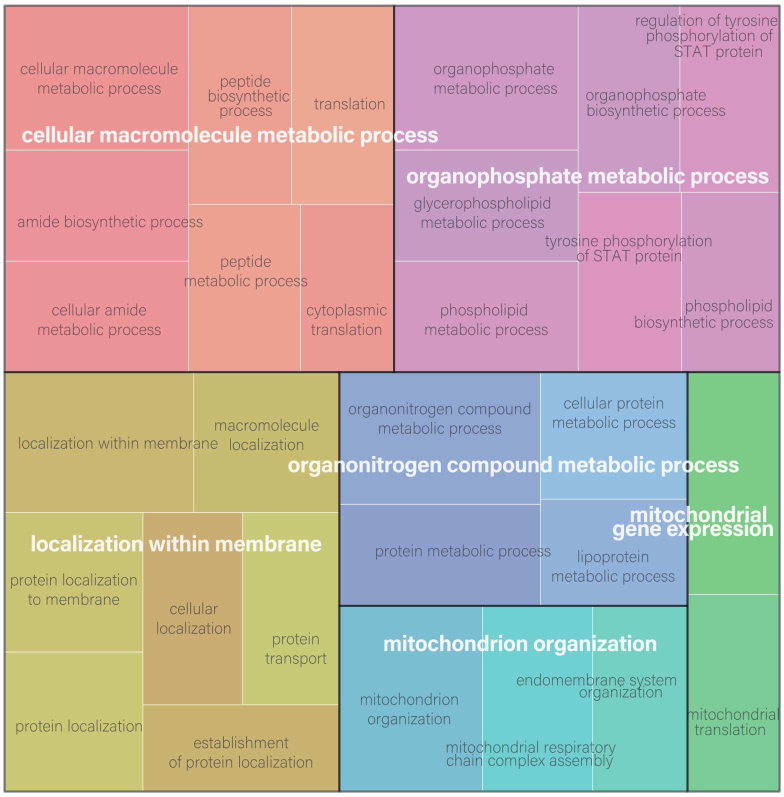

Figure S7

The full representation of **Fig 5a-b**.

a: gene ontology enrichments of unprotected gene functions (ReViGO).

b: Gene ontology enrichments of protected gene functions (ReViGO).

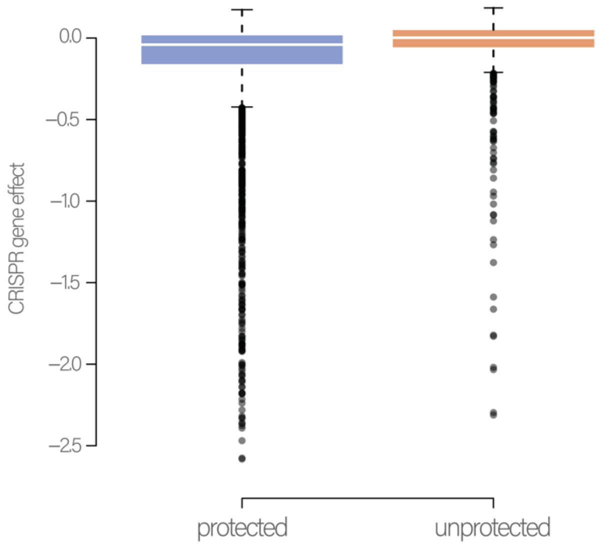

Figure S8

CRISPR gene effect for protected and unprotected genes (protected: n = 633, unprotected: n = 1205; boxplot without outliers in **Fig. 5c**). The box represents the 1st to 3rd quartile with the median marked by a horizontal line. Source data are provided as a Source Data file.

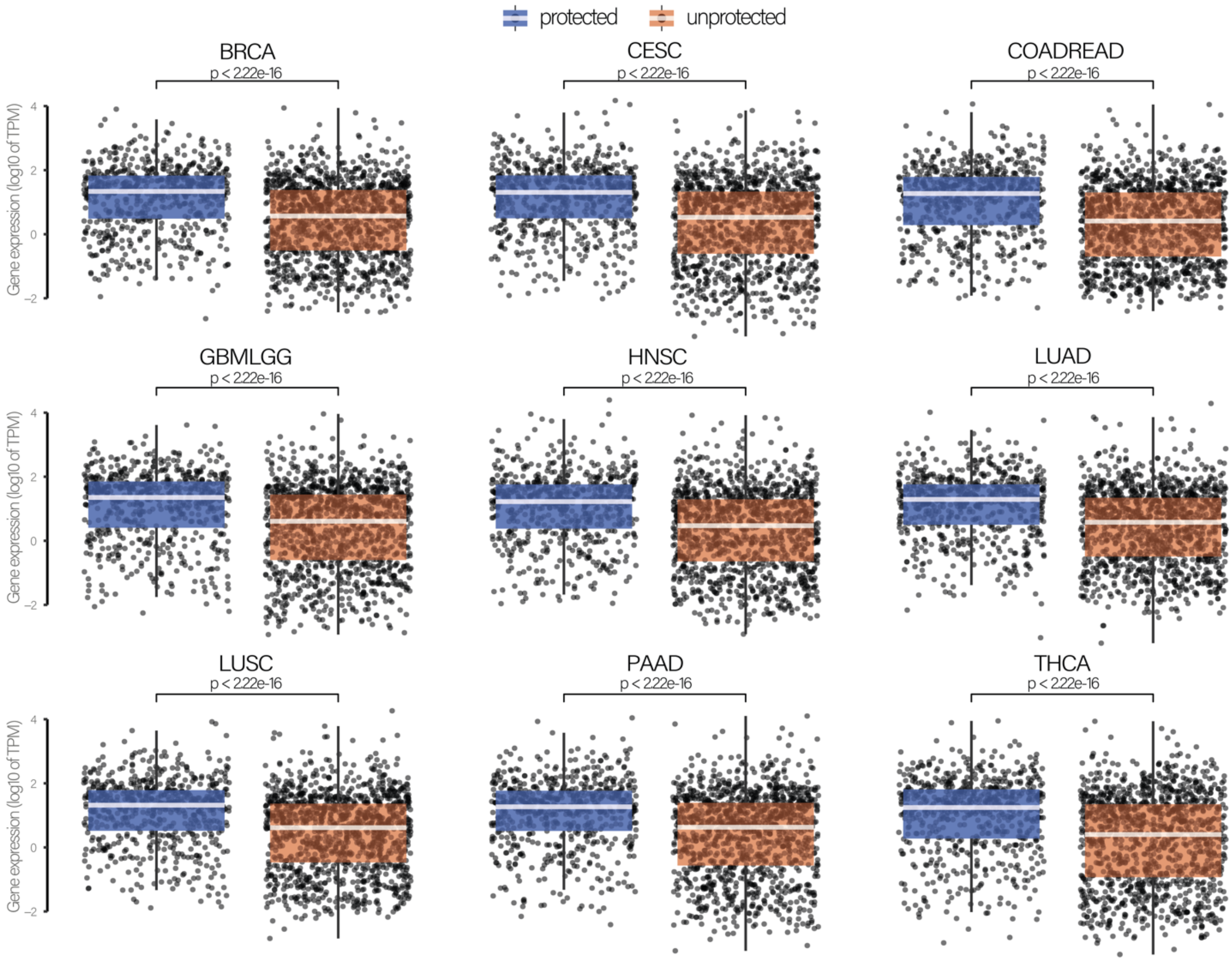

Figure S9

Mean expression of genes within the protected and unprotected gene sets across different cancer types. P-values were determined by a two-sided Wilcoxon test (protected: n = 633, unprotected: n = 1205). The box represents the 1st to 3rd quartile with the median marked by a horizontal line. Lung Adenocarcinoma (LUAD); Breast invasive Carcinoma (BRCA); Lung Squamous cell Carcinoma (LUSC); Glioblastoma multiforme, Brain Lower Grade Glioma (GBMLGG); Thyroid carcinoma (THCA); Colon Adenocarcinoma and Rectum Adenocarcinoma (COADREAD); Pancreatic adenocarcinoma (PAAD); Cervical Squamous cell Carcinoma and Endocervical Carcinoma (CESC); Head and Neck Squamous Carcinoma (HNSC).
