# Supplementary material for: Cancer genomes tolerate deleterious coding mutations through somatic copy number amplifications of wild-type regions
Source: Nat Commun. 2023 Jun 16;14:3594. doi: 10.1038/s41467-023-39313-8 (PMC10276008; doi:10.1038/s41467-023-39313-8)
Supplement: Supplementary file 3 — Description of Additional Supplementary Files [file 41467_2023_39313_MOESM3_ESM.pdf]

Description of Additional Supplementary Files

Supplementary Dataset 1: μ score and amplification frequency for each tumor type at 1 and 36 Mbp and arm-level.

Supplementary Dataset 2: aggregation estimates computed with TANGO.

Supplementary Dataset 3: protected and unprotected genes, gene onthology analysis and CRISPR common essential and non-essential.

Supplementary Dataset 4: number of genes and mutations in each subset of Fig. 2.
